# Systematic review indicates postnatal growth in term infants born small‐for‐gestational‐age being associated with later neurocognitive and metabolic outcomes

**DOI:** 10.1111/apa.13868

**Published:** 2017-05-15

**Authors:** Esther Castanys‐Muñoz, Kathy Kennedy, Eurídice Castañeda‐Gutiérrez, Stewart Forsyth, Keith M. Godfrey, Berthold Koletzko, Susan E. Ozanne, Ricardo Rueda, Marieke Schoemaker, Eline M. van der Beek, Stef van Buuren, Ken K. Ong

**Affiliations:** ^1^ Abbott Nutrition R&D University Science Park Granada Spain; ^2^ UCL Great Ormond Street Hospital Institute of Child Health London UK; ^3^ Nestlé Research Center Lausanne Switzerland; ^4^ DSM‐Martek Division Dundee UK; ^5^ MRC Lifecourse Epidemiology Unit and NIHR Southampton Biomedical Research Centre University of Southampton and University Hospital Southampton NHS Foundation Trust Southampton UK; ^6^ Ludwig‐Maximilians‐Universität Munich Dr. von Hauner Children's Hospital University of Munich Medical Center München Germany; ^7^ Metabolic Research Laboratories & MRC Metabolic Diseases Unit Institute of Metabolic Science University of Cambridge Cambridge UK; ^8^ Abbott Nutrition R&D Granada Spain; ^9^ Mead Johnson Pediatric Nutrition Institute Nijmegen The Netherlands; ^10^ Nutricia Research Danone Nutricia Early Life Nutrition Utrecht The Netherlands; ^11^ Department of Pediatrics University Medical Center Groningen Groningen The Netherlands; ^12^ Netherlands Organisation for Applied Scientific Research TNO Leiden The Netherlands; ^13^ University of Utrecht Utrecht The Netherlands; ^14^ MRC Epidemiology Unit University of Cambridge Cambridge UK

**Keywords:** Adiposity, Insulin resistance, Neurodevelopment, Postnatal growth, Small for gestational age

## Abstract

We systematically reviewed papers published in English between 1994 and October 2015 on how postnatal weight gain and growth affect neurodevelopment and metabolic outcomes in term‐born small‐for‐gestational‐age (SGA) infants. Two randomised trials reported that enriched infant formulas that promoted early growth also increased fat mass, lean mass and blood pressure (BP), but had no effect on early neurocognitive outcomes. Meanwhile, 31 observational studies reported consistent positive associations between postnatal weight gain and growth with neurocognitive outcomes, adiposity, insulin resistance and BP.

**Conclusion:** Few intervention studies exist, despite consistent positive associations between early growth and neurocognition in term‐born SGA infants.

**Abbreviations**
AGA: Appropriate for gestational age
BMI: Body mass index
BP: Blood pressure
RCT: Randomised controlled trial
SGA: Small for gestational age

**Key Notes**

- This systematic review covering 1994–2015 focused on how postnatal weight gain and growth affect neurodevelopment and metabolic outcomes in term‐born small‐for‐gestational‐age infants.
- Two randomised trials reported that enriched infant formulas that promoted early growth also increased fat mass, lean mass and blood pressure (BP), but not neurocognitive outcomes.
- Observational studies reported consistent positive associations between postnatal weight gain and growth with neurocognitive outcomes, adiposity, insulin resistance and BP.

## Introduction

Many infants who are small at birth tend to gain weight more rapidly during the early postnatal period. This catch‐up growth may be advantageous in certain populations of low‐birthweight children, with potential short‐term beneficial effects on survival and longer‐term benefits on cognitive development and stature 1. Conversely, accelerated postnatal weight gain and catch‐up growth has also been associated with an increased risk of adiposity and metabolic disease later in life 2, 3, 4. A wealth of evidence indicates that events in early life can influence the risk of later disease. It is well established that developmental plasticity allows organisms to adapt their phenotype through epigenetic, metabolic and, or, anatomical processes, in response to certain stimuli 5. However, if the actual conditions do not match the ones predicted, the adaptation can have an adverse impact on long‐term health, leading to disease 6. Associations have been widely observed between low birthweight and later noncommunicable diseases, such as type 2 diabetes 7. Evidence from animal and human models has led to the suggestion that those associations with low birthweight are mediated, in part, by rapid postnatal weight gain and catch‐up growth leading to excessive adiposity 8, 9.

Low birthweight may be due to a number of different reasons, including intrauterine growth restriction and preterm birth. We previously reviewed the relationship between postnatal growth rates, cognitive outcomes and the risk of disease in preterm infants 10. That review concluded that more research was needed to understand how to optimise growth in preterm infants and to achieve neurocognitive benefits while minimising the risk of later disease. However, the effects of rapid postnatal weight gain need to be considered separately in different populations of low‐birthweight infants, as the benefits and the risks may differ between preterm infants and full‐term infants born small for gestational age (SGA). In preterm infants, catch‐up growth may be beneficial for neurodevelopment 11, 12, particularly if catch‐up in length and height is achieved, and this may outweigh the potential long‐term metabolic costs. Being born SGA also constitutes a risk factor for the development of diseases later in life, as these infants are likely to experience accelerated postnatal weight and body fat gain 13. SGA is commonly defined as birthweight and, or, length that is at least two standard deviation scores below the mean for gestational age 14, although some publications set the cut‐off below the third, fifth or 10th percentile 15, 16, 17. The minus two standard deviation scores definition is likely to capture the majority of infants with impaired foetal growth. While numerous studies have identified associations between early postnatal weight gain or body fat gain with later disease risk factors, not many have specifically evaluated populations of full‐term SGA infants.

The aim of this review was to identify and summarise the published evidence on postnatal weight gain and growth in term‐born SGA infants, with regard to the potential neurodevelopmental benefits and adverse metabolic outcomes. It also attempts to identify critical postnatal windows during which growth might influence these outcomes.

## Methods

A systematic search of electronic databases was performed using ProQuest Dialog. The Medline and EMBASE databases were combined to identify studies published between 1994 and October 2015 that reported associations between postnatal growth patterns and later metabolic and neurocognitive outcomes in term infants born SGA. Relevant studies prior to 2005 were obtained from the review by Baird 18.

**Figure 1:**
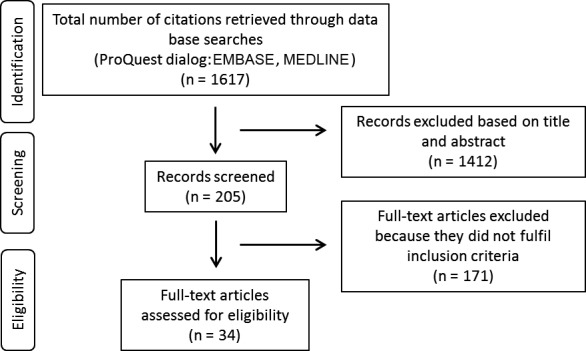
Flow chart of search strategy and selection of relevant studies.

Search terms representing the following categories were combined: (i) the population, such as term infants or SGA, (ii) postnatal growth, including catch‐up growth, early growth and failure to thrive, (iii) outcomes related to metabolic diseases such as adiposity, insulin resistance and cardiovascular risk or to neurocognitive development, such as neurodevelopment and the intelligence quotient. One author (EC‐M) carried out the search and assessed the titles and abstracts to identify relevant studies. Then, the full‐text papers of the potentially eligible studies were screened and selected for inclusion in the systematic review if they met the specific selection criteria. An additional hand search of reference lists of relevant and related papers was made to ensure a complete collection. Around 200 full‐text papers were reviewed (Fig. 1).

### Inclusion and exclusion criteria

The search terms for the study population were the following: small for gestational age, SGA, intrauterine growth restriction, IUGR, growth restricted, growth retarded, low birthweight and not preterm. Only studies on infants born at term were included. Studies that also reported preterm data were only included if the findings were presented separately.

With regard to exposure, special attention was given to using a definition of growth that considered changes in weight and, or, height between at least two time points, such as birth to 16 weeks, rather than measurements of body size at one specific age. Growth was also accepted if reported as absolute velocity or as a change in standard deviation scores. In the latter case, the growth reference used needed to be reported. Studies were accepted if the outcome was assessed at the same age as the second growth measurement or later.

The metabolic outcomes that we considered were related to body composition – such as overweight, obesity and adiposity – insulin resistance, glucose tolerance or cardiovascular risk, including the blood lipid profile and blood pressure (BP). The neurodevelopmental outcome measures included: neurodevelopment, cognition, intelligence quotient, motor function and behaviour. No limit was applied regarding the subjects’ ages at the time of the outcome assessment. The earliest was four months and the latest was in 21‐year‐old young adults.

Both intervention and observational studies were accepted. Intervention studies were selected if the intervention influenced postnatal growth and the study assessed at least one of the outcomes mentioned above.

The search was limited to studies in humans and original papers published in English. Studies were excluded if the body size was only reported at one time point. Concerns about the eligibility of specific studies were resolved by seeking a second opinion from another author (KK). When further assessment was needed, consensus was reached by other authors (ECG, KG, KKO). Only published data were considered, and no further data were requested from study authors. Additional studies suggested by co‐authors for possible inclusion were considered, but no new eligible studies were identified this way.

One author (EC‐M) was responsible for the data extraction, tabulation and preliminary interpretation of the 34 individual reports, of which six examined neurodevelopmental outcomes and 28 examined metabolic outcomes. As 16 (47%) of these papers reported several outcomes of interest, they were used more than once.

Data were retrieved using specifically designed forms that included the study design, timing and nature of the exposure, number of participants and age at follow‐up measurements. A summary result indicator was attributed to each study, whether it reported a positive effect and, or, the association between postnatal growth and the outcome measure. A summary result indicator was attributed to each study, whether it reported a positive effect and, or, association between postnatal growth and the outcome measure: (++) statistically significant positive association; (+) non‐significant positive trend; (0) no association; (−) non‐significant inverse trend; and (−−) significant inverse association.

A quantitative summary of the data by meta‐analysis could not be undertaken due to the heterogeneity of the studies in terms of the measurement of the exposure, outcome variables and timing of measurements between studies. A descriptive methodology was chosen, and the results are presented as a narrative synthesis.

To ensure the study quality, three authors (EC‐M, KK, KKO) independently assessed the methodological quality of the observational studies using the Newcastle–Ottawa Scale 19. This assessment evaluates study quality in relation to the control of confounding variables, adequate sample size, minimisation of selection bias and definition of exposure. Studies were given scores: the selection of the study groups could score a maximum of four points, the comparability of the study groups could score a maximum of two points, and the ascertainment of the outcome could score a maximum of three points. The highest possible score was nine.

## Results

We included 34 papers on postnatal weight gain and growth in term infants born SGA (summarised in Tables 1 and 2): most of these (n = 31) described observational studies, with only three papers from the same group describing different outcomes from two intervention studies 20, 21, 22. With regard to study quality, the majority were rated as moderate or high quality. Only five studies were considered to be of low quality, with a score of four or less. These are noted in Tables 1 and S1a–c.

**Table 1:** Postnatal growth in full‐term SGA infants related to later neurocognitive outcomes

WT gain	Timing of exposure	N SGA	Age at outcome	Summary result	Comments	Adjustment
Randomised controlled trials						
Morley 2004^M^ 20	B‐9 mo (Nutrient‐enriched formula)	299	9 mo 18 mo	– (9 mo) 0 (18 mo)	At 9 mo, enriched formula group had lower developmental quotient than the standard formula group At 18 mo, no difference was seen in Bayley MDI or PDI scores	Sex, age, maternal education, social class
Observational studies (n = 5)						
Pylipow 2009^M^ 27	B‐4 mo (WT gain)	503	7 y	− (inverted J‐shape)	Both fast or slow WT gain to age 4 mo associated with lower WISC	Sex, race, SES
Jensen 2015^M^ 25	B‐3 mo B‐17 y (HT gain, HC gain)	47	17 y	+ (B‐3 mo) ++ (B‐17 y)	HT gain to age 3 mo showed a positive trend with IQ HT gain to age 17 y was positively associated with IQ	Sex, age, parental SES, maternal age
Horta 2009^M^ 24	B‐20 mo (WT gain) 20–42 mo (WT gain)	1822	18 y	++ (B‐20 mo) 0 (20–42 mo)	WT gain to age 20 mo, but not between 20 and 42 mo, was positively associated with achieved schooling	Parental education, household assets, SES
Lundgren 2001^M^ 26	B‐18 y (WT gain)	6440	18 y	++	WT gain was positively associated with IP and PP	Birthweight and length
Strauss 2000^M^ 23	B‐5 y (HC gain)	1064	26 y	++	HC gain was positively associated with professional attainment and a positive trend with income	Sex, parental SES, neonatal distress

Reports are ordered by study design and age at outcome assessment.

B, birth; MDI, mental developmental index; PDI, psychomotor developmental index; WT, weight; HT, height or length; IP, intellectual performance; PP, psychological performance; SES, socioeconomic status; WISC, Wechsler intelligence scale for children; HC, head circumference; y, years; mo, months.

Study quality: Low (L), 0–4 points in the Newcastle–Ottawa Scale; moderate (M), 5–7 points; high (H), 8–9 points.

++, statistically significant positive association; +, non‐significant positive trend; 0, no association; −, non‐significant inverse trend; −−, significant inverse association.

**Table 2:** Summary of findings relating postnatal growth in full‐term small‐for‐gestational‐age infants to neurocognitive and metabolic outcomes in the 34 identified papers

Outcomes	Study design	No. of studies	No. of subjects	Summary of findings
Neurocognition	Randomised trial	1	299	Enriched formula from birth to nine months increased infancy weight gain and length growth, but there was no difference in Bayley's score at 18 months
	Observational	5	1482	4/5 studies (n = 979) reported early growth was positively associated with cognition at ages 17 to 26 years. 1/5 study (n = 503) reported both fast growth and slow early growth were associated with lower intelligence quotient at seven years
Body mass index (BMI)	Observational	14	2151	11/14 studies (n = 2031) reported early growth was positively associated with BMI. 3/14 studies (n = 120) reported no association between early growth and later BMI
Per cent body fat	Randomised trial	1	299	Enriched formula increased infancy weight gain and length growth and also increased per cent body fat at ages six to eight years
	Observational	5	1476	2/5 studies (n = 54) reported early growth was positively associated with per cent body fat at ages two to four years. 3/5 studies (n = 1412) reported no association between early growth and later per cent body fat at ages four months to 21 years
Fat and lean mass	Randomised trial	1	246	Enriched formula increased infancy weight gain and length growth and also increased fat mass at ages five to seven years.
	Observational	6	591	5/6 studies (n = 486) reported early weight gain was positively associated with fat mass at ages one to eight years. 1/6 study (n = 106) reported early weight gain was positively associated with lean mass but not fat mass at age 21 years
Insulin resistance	Observational	12	1119	12/18 studies (n = 763) reported weight gain (birth to 7–21 years) was positively associated with insulin resistance. 6/18 studies (n = 356) reported no association between postnatal growth and later insulin resistance
Blood pressure	Randomised trial	1	153	Enriched formula increased infancy weight gain and length growth and also increased the risk of higher blood pressure at six to nine years
	Observational	4	3180	3/4 studies (n = 3074) reported early weight gain was positively associated with blood pressure at ages 6 to 15 years. 1/4 study (n = 106) reported no association between early weight gain with blood pressure at age 21 years
Lipids	Observational	7	585	1/7 study (n = 165) reported early weight gain was positively associated with total cholesterol and triglycerides at age eight years. 6/7 studies (n = 420) reported no association between early weight gain with later lipid profiles

### Neurocognitive outcomes

Only one randomised controlled trial (RCT) 20 promoted early growth in SGA infants using a nutrient‐ and energy‐enriched formula – with higher protein, lipids, carbohydrate and micronutrient contents – and reported neurocognitive outcomes. The enriched formula group had a lower developmental quotient than the standard formula group at the age of nine months and no difference in neurocognitive outcomes measured using the Bayley Mental Development and Psychomotor Development indexes at the age of 18 months (Table 1).

In total, four observational studies comprising 9373 children (Table 1) reported that faster postnatal growth in weight, length and, or, height or head circumference between birth and 20 months to five years was associated with better neurocognitive outcomes at the ages of 17 to 26 years 23, 24, 25, 26. One further study comprising 503 children reported a quadratic association: both low and high weight gains between birth and nine months were associated with lower cognitive function at the age of seven years 27. Most observational studies adjusted for a variety of potential confounding factors, although none adjusted for maternal intelligence quotient. Only one study compared SGA infants versus an appropriate for gestational age (AGA) control group: this study found lower cognitive function in SGA than AGA children 27.

### Adiposity

One paper reported two RCTs comprising 545 children 22. Both RCTs showed that nutrient‐ and energy‐enriched formulas, which promoted early growth, also increased total body fat mass in SGA‐born children at the age of five to seven years, along with a trend for increased total body lean mass (Table S1a).

There were 18 independent observational studies that reported on the association between postnatal growth and some measures of adiposity: Table S1a–c show the findings stratified by outcome measure. Faster postnatal growth, measured as weight, length and, or, height or body mass index (BMI) between birth and one to eight years were positively associated with BMI at the ages of one to 21 years 14, 16, 28, 29, 30, 31, 32, 33, 34, 35, 36, as reported in 11 studies comprising 784 children. However, three further studies comprising 120 children reported no association between postnatal growth, measured as the weight between birth and two to seven years and BMI at the ages of two to seven years 37, 38, 39. Faster postnatal growth, measured as the weight gain between birth and four to seven years, was positively associated with the percentage of body fat at the ages of two to 11 years in three studies comprising 1311 children 34, 35, 38, whereas two further studies comprising 165 children reported no association between postnatal growth, measured as the weight gain between birth and one year of age, and the percentage of body fat at the ages of one to 21 years 40, 41. Furthermore, faster growth, measured as the weight gain between birth and two to seven years, was positively associated with total body fat mass at the ages of two to seven years in two studies comprising 247 children 36, 39. However, another study comprising 106 children showed no association between postnatal growth, in terms of the weight gain between birth and 21 years, and total body fat mass at the age of 21 years 42. Using total body lean mass at the ages of seven to 21 years as an outcome measure, a positive association was found with faster growth in terms of weight gain between birth and seven to 21 years in two studies comprising 162 children 39, 42. In contrast, another study comprising 29 children reported an inverse relationship between weight gain from birth to two years and total body lean mass at the age of two to four years 38. Meanwhile, in two studies comprising 79 children, abdominal fat mass at the ages of one to four years was positively associated with faster growth, measured as the weight gain between birth and two to four years 38, 43. Finally, five of the 11 observational studies, which comprised 1513 children including AGA controls, reported that the SGA group had significantly lower total body adiposity measured as the percentage of body fat and, or, BMI compared to the AGA group at the ages of one to seven years 15, 28, 30, 33, 34. Conversely, two studies comprising 194 children reported that those born SGA had significantly higher adiposity, measured as a percentage of body fat, and a higher subscapular‐to‐triceps skinfold ratio compared to the AGA group at the ages of two to eight years 38, 44.

### Insulin resistance

No RCTs included any measure of insulin resistance as the outcome (Table S1a), but 12 studies comprising 763 children reported that faster postnatal growth, measured as the weight gain between birth and eight to 21 years, was positively associated with insulin resistance at the ages of one to 21 years, using the homeostatic model assessment for insulin resistance, fasting insulin, oral glucose tolerance test, intravenous glucose tolerance test and clamp 15, 16, 29, 33, 35, 38, 39, 41, 44, 45, 46, 47. A further six studies comprising 356 children reported no association between postnatal growth, measured as weight, height or BMI between birth to eight years, and insulin resistance at the ages of one to eight years, using the homeostatic model assessment for insulin resistance or fasting insulin 17, 30, 31, 37, 40, 43 (Table S1b). Positive associations were reported by eight studies with adjustments for current adiposity and four studies without. Of the 13 studies that included AGA controls, 10 studies reported that the SGA group had significantly higher insulin resistance, measured as fasting insulin, oral glucose tolerance test, intravenous glucose tolerance test and clamp, than the AGA group at the ages of one to 21 years 15, 16, 29, 33, 35, 38, 41, 45, 46, 47.

### Blood pressure

Only one RCT, comprising 153 children, reported that a nutrient‐ and energy‐enriched formula given from the age of four days to nine months promoted weight gain and increased the risk of high BP at six to nine years 21 (Table S1c). In four observational studies comprising 3180 children, including an observational study among breastfed children 21, faster postnatal growth measured as the weight gain between birth and two to eight years was positively associated with a higher risk of high BP at the ages of seven to 15 years 21, 44, 48, 49. In contrast, one further study comprising 106 children reported no association between weight gain at birth to 21 years with BP at the age of 21 years. Instead, that study reported a positive association between weight gain and carotid intima media thickness at 21 years of age 42. None of the four studies that included AGA controls reported that the SGA group had significantly higher BP than the AGA group at the ages of seven to 21 years 42, 44, 48, 49.

### Serum lipid concentrations

No RCTs were reported with blood lipid levels as the outcome, but seven observational studies reported on the association between postnatal weight gain or growth and blood lipid levels (Table S1c) and these studies varied widely in measures of early growth and lipid parameters. Only one study comprising 165 children reported that faster weight gain between birth to eight years was positively associated with higher total cholesterol and triglycerides at the age of eight years 44. In contrast, a further five studies comprising 364 children reported no association between postnatal growth between the ages of zero and one to eight years and lipid levels in terms of total cholesterol, low‐density lipoprotein, high‐density lipoprotein or triglycerides at the ages of one to eight years 15, 17, 31, 37, 45. Furthermore, three studies comprising 189 children reported that faster height or length gain between birth and five to eight years was negatively associated with total cholesterol, low‐density lipoprotein and high‐density lipoprotein 17, 31, 50. Only one of the seven studies that included an AGA control group reported that the SGA group, particularly those with poor height growth, had significantly higher total cholesterol compared to the AGA group at the age of 12 years 50.

## Discussion

This review of postnatal weight gain and growth in relation to neurocognitive and metabolic outcomes in term‐born SGA children only found two RCTs that met the eligibility criteria. The same RCT was reported with differing outcomes in three papers 20, 21, 22, and a second RCT was also described by the Singhal et al. paper 22. These trials showed that nutrient‐ and energy‐enriched formulas that promoted early growth increased fat mass and also demonstrated some increases in lean body mass and increased BP. There was no benefit on neurocognitive outcomes.

The observational studies generally reported consistent associations between postnatal weight gain or growth with neurocognitive outcomes, adiposity, insulin resistance and BP. Postnatal weight gain appeared to be beneficial for neurocognitive outcomes, but seemed to have adverse effects on adiposity and related markers of metabolic health. However, it is important to consider the metabolic risks that SGA individuals face in the context of the general population. Compared to AGA controls, SGA groups had higher insulin resistance, but lower total adiposity – albeit most commonly assessed by BMI – and no evidence for higher BP.

Most of the observational studies were of moderate to high quality. The four studies rated as low quality were distributed across the outcome categories and their exclusion would have had little or no impact on the conclusions. We were unable to perform meta‐analyses due to the high variability in age at outcome and the paucity of comparable outcome measures. However, we found consistent observational evidence for most outcomes.

Only one study compared SGA to an AGA control group with regard to neurocognitive outcomes 25. However, one other review reported lower verbal and performance intelligence quotient scores in school‐age children born SGA 51 and neuroimaging studies have shown early microstructural changes related to altered neurobehavioral outcomes 52. We found consistently positive associations between postnatal growth and neurocognitive outcomes in four of the five observational studies included in the review 51. Interestingly, the only RCT included in this review reported a negative effect of growth induced by nutrient‐ and energy‐enriched formula feeding on developmental scores at nine months in just girls, but no differences between nutritional groups at 18 months. The discordance between the RCT and the observational studies in the review 51 may have been due to the early age at which neurocognitive outcomes were measured in the RCT – nine and 18 months – compared to the observational studies at seven to 26 years. Alternatively, residual confounding factors may have remained in the observational studies. For example, comorbidities that arose during the neonatal period might have had an adverse impact on both growth and neurocognition.

For adiposity and metabolic outcomes, most studies considered the period of postnatal weight gain exposure as birth to the age at outcome. In such analyses, the influence of growth would be dominated by the current body size. This was the case for: 11 of 14 studies for BMI; three of five studies for per cent body fat; four of six studies for fat mass; 15 of 18 studies for insulin resistance, and two out of four studies for BP, although in all cases the remaining studies all reported significant positive associations. Hence, the exclusion of such studies did not materially alter the conclusions. However, there were insufficient data with discrete periods of postnatal weight gain to estimate the presence of critical, early windows of weight gain or growth associated with later outcomes.

Despite the consistent evidence for a positive association between postnatal weight gain and adiposity, SGA individuals continued, on average, to have less adipose than AGA individuals. With the exception of two observational studies that measured abdominal fat, adipose tissue distribution was not evaluated among the outcomes. The possibility that SGA children accumulate greater visceral adiposity than AGA children without greater BMI or total body fat cannot be discarded.

Only one of the seven observational studies reported a positive association between postnatal weight gain or growth and blood total cholesterol levels. However, these studies only included children only up to age 12 years old and we note that total cholesterol is an imprecise marker in cardiovascular disease risk in children due to their higher proportion of high‐density lipoprotein to low‐density lipoprotein cholesterol.

The observational studies were set across diverse geographical regions. This was a strength of the evidence base as most full‐term SGA infants are born in low‐income and middle‐income countries 53 and these babies face higher risks of postnatal growth failure. It is possible that it is not weight gain that predicts metabolic and disease outcome, but rather the infancy gains in lean versus fat mass and, or, changes in fat mass distribution. However, few studies to date have related changes in infancy body composition to later outcomes.

### Interpretation for policy and recommendations for future studies

In general, SGA infants face an increased risk of short stature, with average adult heights being approximately one standard deviation score below the mean population height 54. However, faster postnatal growth and weight gain can lead to taller adult heights, but this study did not review that factor 54. Although the evidence for a similar positive impact on neurocognitive outcomes is relatively sparse, the potential for such long‐term benefits on height and neurocognition makes the promotion of infancy weight gain an appealing policy. On the other hand, insulin resistance is higher in SGA catch‐up children than in the general population and this finding is supported by consistent evidence on the association between low birthweight and type 2 diabetes 7.

Regarding long‐term cardiovascular disease risks, prenatal growth restriction can result in prenatal circulatory adaptations and altered heart and vascular tree development 55 and one study reported that weight gain over the long period from birth to 21 years was positively associated with carotid intima media thickness at 21 years 42. We found consistent experimental and observational evidence linking postnatal weight gain and growth to higher BP. Nevertheless, none of the studies reported higher BP in SGA versus AGA groups and, as discussed above, the evidence on lipid outcomes was inconsistent. Thus, the relevance of postnatal growth to cardiovascular disease risk in SGA individuals remains unclear.

Establishing the optimal growth patterns in term‐born SGA infants to minimise short‐term and long‐term risks is crucial. It has been proposed that reaching around the 30th weight percentile in the first postnatal months and around the 50th percentile by the age of seven may prevent the risks of adverse outcomes 56. However, that hypothesis was only based on observational data and it remains a challenge to safely promote infancy growth in SGA, let alone attain such a narrow growth target. Furthermore, whether such growth patterns are appropriate across settings that differ in exposure to infections and risk of malnutrition is unknown.

## Conclusion

This review summarises the published evidence on postnatal weight gain and, or, growth in term‐born SGA infants, regarding the potential neurodevelopmental benefits and adverse metabolic outcomes. Only two RCTs were identified, including one that reported on neurocognitive outcomes and found no effect. Overall, 31 observational studies did report consistent positive associations between postnatal weight gain and, or, growth with neurocognitive outcomes, adiposity, insulin resistance and BP.

In conclusion, our systematic review highlights hard it was to find RCTs that focused on promoting growth in full‐term SGA infants. There is great potential for such interventions to have long‐term benefits on height and neurocognition without significant negative effects on metabolic health, but there remains a substantial knowledge gap on the impact of nutritional interventions on growth in this population, especially in low‐income and middle‐income settings. Further observational studies that include repeated postnatal anthropometric measurements would allow us to identify possible critical windows of growth associated with differing health outcomes and more studies assessing neurocognitive outcomes are needed. Finally, future prospective studies should assess the potential contribution of body composition, rather than total body weight gain on later outcomes. It is plausible that the potential benefits of postnatal growth on neurocognitive and other outcomes are related to gains in lean body mass, whereas gains in body fat mass, in particular central fat, might predict insulin resistance and related adverse metabolic and health outcomes.

## Conflicts of interest

Keith Godfrey has received reimbursement for speaking at conferences sponsored by companies selling nutritional products and is part of an academic consortium that has received research funding from Abbott Nutrition, Nestec and Danone. Berthold Koletzko is a member of the National Breastfeeding Committee. The Ludwig‐Maximilians‐Universität München and its employee Berthold Koletzko have received support for scientific and educational activities by companies marketing nutritional products, predominantly as part of publically funded research projects with support of the European Commission or German governmental research support. The other authors declare no conflict of interest.

## Funding

The expert group received funding from the ILSI Europe Early Nutrition and Long‐Term Health (formerly Metabolic Imprinting) Task Force. Industry members of this task force are listed on the ILSI Europe website at http://ilsi.eu/task-forces/nutrition/early-nutrition-and-long-term-health/. Experts are not paid for the time spent on this work; however, the nonindustry members within the expert group received a small compensatory sum (honoraria) and travel support from the Early Nutrition and Long‐Term Health Task Force to attend meetings to discuss the review.

## Supporting information


**Table S1** Postnatal growth in full‐term SGA infants related to: a) adiposity; b) insulin resistance; c) blood pressure and d) lipid profiles. Studies are ordered by study design and age at outcome assessment.
