# Supplementary material for: Systematic review indicates postnatal growth in term infants born small‐for‐gestational‐age being associated with later neurocognitive and metabolic outcomes
Source: Acta Paediatr. 2017 May 15;106(8):1230–8. doi: 10.1111/apa.13868 (PMC5507303; doi:10.1111/apa.13868)
Supplement: Supplementary file 1 — Table S1 Postnatal growth in full‐term SGA infants related to: a) adiposity; b) insulin resistance; c) blood pressure and d) lipid profiles. Studies are ordered by study design and age at outcome assessment. [file APA-106-1230-s001.docx]

Table S1: Postnatal growth in full-term SGA infants related to: a) adiposity; b) insulin resistance; c) blood pressure and d) lipid profiles. Studies are ordered by study design and age at outcome assessment.

| **a) Adiposity** | **Timing of exposure**  **(nature)** | **N**  **SGA** | **Age at outcome** | **Summary result** | **SGA vs. AGA controls** | **Comments** |
| --- | --- | --- | --- | --- | --- | --- |
| **Randomised controlled trials** | | | | | | |
| Singhal 2010^M^  (22) | 1. B-9mo  2. B-6mo  (Nutrient enriched formula) | 1. 299  2. 246 | 1. 6-8y  2. 5-7y | ++  (% body fat)  0 (BMI)  ++ (Total fat mass) |  | In both studies, the enriched formula increased WT and HT gains and later fat mass  Faster WT and HT growth were associated with later fat mass |
| **Observational studies (n=14)** | | | | | | |
| Soto 2003^M^  (15) | B-1y  (WT, HT gain) | 85 | 1y | ++  (BMI) | --  (BMI) | WT gain, but not HT gain, was positively associated with BMI |
| Iniguez 2004^L^ (28) | B-2y  (WT gain) | 65 | 2y | ++  (BMI) | --  (BMI) | WT gain was positively associated with BMI |
| Taal 2013^M^  (36) | B-2y, 2-4y  (WT, HT) | 191 | 2y, 4y | ++  (BMI at 4y) |  | WT gain was positively associated with fat mass at 2y and BMI at 4y |
| Ibañez 2006^M^ (38) | B-2y  2-4y  (WT gain) | 29 | 2y, 4y | 0  (BMI) |  | WT gain between 0-2y was not associated with higher BMI (**adjusted for height**) |
| Mohn 2007^L^ (35) | B-4y  (WT gain) | 35 | 4y | ++  (BMI) | 0  (BMI) | WT gain was positively associated with BMI |
| Deng 2011^M^  (33) | B-6y  (WT, HT) | 67 | 6y | +  (BMI) | --  (BMI) | Trend to higher BMI in children with catch-up growth. |
| Uçar 2014^L^  (29) | B-6.5y  (WT, HT gain) | 31 | 6.5y | ++  (BMI) |  | WT gain was positively associated with BMI in girls |
| Miras 2010^M^ (30) | B-6.5y  (WT gain) | 49 | 6.5y | ++  (BMI) | --  (BMI) | WT gain was positively associated with BMI |
| Evagelidou 2007^M^ (37) | B-7y  (WT gain) | 35 | 7y | 0  (BMI) |  | WT gain was not associated with BMI |
| Ibañez 2009^M^ (39) | B-7y  (WT gain) | 56 | 7y | 0  (BMI) |  | WT gain was not associated with BMI |
| Torre 2008^M^ (31) | B-8y  (WT, HT, BMI) | 52 | 8y | ++  (BMI) | 0  (BMI) | WT gain was positively associated with BMI |
| Cianfarani 2003^M^ (16) | B-8.5y  (HT gain) | 82 | 8.5y | ++  (BMI) |  | HT gain was positively associated with BMI |
| Kramer 2014^M^ (34) | B-6mo  6mo-6.5y  6.5y-11.5y  (WT gain) | 1247 | 6mo, 6.5y, 11.5y | +  (BMI) | --  (BMI) | WT gain was not significantly associated with adiposity (**adjusted for parental height**) |
| Ezzahir 2005^M^ (32) | B-1y  B-2y  B-6y  (BMI gain) | 127 | 21y | ++  (BMI) | +  (BMI) | BMI gain, especially after the first year of life, was positively associated with adiposity |
|  | | | | | | |
| **Observational studies (n=5) - % Body fat** | | | | | | |
| Beltrand 2009^L^ (40) | B-4mo  4mo-1y  (WT gain) | 94 | 4mo, 1y | 0  (% body fat) |  | WT gain was not associated with % body fat |
| Ibañez 2006^M^ (38) | B-2y  2-4y  (WT gain) | 29 | 2y, 4y | ++ (% body fat) | ++ ( % body fat) | WT gain between 0-2y was positively associated with adiposity and fat gains between 2-4y (**adjusted for height**) |
| Mohn 2007^L^ (35) | B-4y  (WT gain) | 35 | 4y | ++  (% body fat) | 0  (% body fat) | WT gain was positively associated with BMI and % fat mass |
| Kramer 2014^M^ (34) | B-6mo  6mo-6.5y  6.5y-11.5y  (WT gain) | 1247 | 6mo, 6.5y, 11.5y | +  (% body fat) | --  (% body fat) | WT gain was not significantly associated with adiposity (**adjusted for parental heights**) |
| Leunissen 2008^H^ (41) | B-21.5y  (WT gain) | 71 | 21.5y | 0 (% body fat) |  | WT gain was not significantly associated with adiposity (**adjusted for height**) |
|  | | | | | | |
| **Observational studies (n=6) - Fat or lean mass** | | | | | | |
| Amador-Licona 2007^L^ (43) | B-1y  (WT gain) | 44 | 1y | ++  (Abdo. fat) |  | WT gain was positively associated with abdominal fat |
| Taal 2013^M^  (36) | B-2y, 2-4y  (WT, HT) | 191 | 2y, 4y | ++  (Total fat mass at 2y) |  | WT gain was positively associated with fat mass at 2y and BMI at 4y |
| Ibañez 2006^M^ (38) | B-2y  2-4y  (WT gain) | 29 | 2y, 4y | ++ (Abdo. fat)  - - (Lean mass) | ++ (Abdo. fat)  - - (Lean mass) | WT gain between 0-2y was positively associated with adiposity and fat gains between 2-4y (**adjusted for height**) |
| Ibañez 2009^M^ (39) | B-7y  (WT gain) | 56 | 7y | ++ (Total fat &  Lean mass) |  | WT gain was positively associated with fat and lean mass, and also subcutaneous and visceral fat |
| Bavdekar 1999^M^ (44) | B-8y  (WT gain) | 165 | 8y | ++  (SS/TR) | ++  (SS/TR) | WT gain was positively associated with fat mass |
| Leunissen 2012^M^ (42) | B-21y  (WT gain) | 106 | 21y | 0 (Total fat mass)  ++ (Total lean mass) |  | WT gain was positively associated with lean mass, but not fat mass |

| **b) Insulin resistance** | **Timing of exposure (nature)** | **N SGA** | **Age at outcome** | **Summary result** | **SGA vs. AGA controls** | **Comment** |
| --- | --- | --- | --- | --- | --- | --- |
| **Observational studies (n=18)** | | | | | | |
| Amador-Licona 2007^L^ (43) | B-1y  (WT gain) | 44 | 1y | 0  (HOMA-IR) |  | WT gain was not associated with IR |
| Beltrand 2009^L^  (40) | B-4mo  4mo-1y  (WT gain) | 94 | 1y | 0  (Fasting insulin) |  | WT gain was not associated with IR |
| Soto 2003^M^  (15) | B-1y  (WT, HT gain) | 85 | 1y | ++  (Fasting insulin) | ++  (Fasting insulin) | Catch up growth was associated with higher IR (**adjusted for BMI**) |
| Mericq 2005^M^  (47) | B-3y  (WT gain) | 55 | 3y | ++  (HOMA-IR) | ++  (HOMA-IR) | WT gain was positively associated with IR |
| Ibañez 2006^M^  (38) | B-2y  2-4y  (WT gain) | 29 | 2y, 3y, 4y | ++ (HOMA-IR @4y) | -- (HOMA-IR @2y)  ++ (HOMA-IR @4y) | WT gain between 0-2y was positively associated with change in IR between 2-4y |
| Mohn 2007^L^  (35) | B-4y  (WT gain) | 35 | 4y | ++  (HOMA-IR) | ++  (HOMA-IR) | WT gain was positively associated with IR |
| Deng 2012^M^  (45) | B-6y  (HT, BMI gain) | 111 | 6y | ++  (HOMA-IR) | ++  (HOMA-IR) | HT & BMI gains were positively associated with IR  (**adjusted for BMI**) |
| Deng 2011^M^  (33) | B-6y  (HT gain) | 67 | 6y | ++  (HOMA-IR) | ++  (HOMA-IR) | HT gain was positively associated with IR (**adjusted for BMI**). |
| Miras 2010^M^  (30) | B-6.5y  (WT gain) | 49 | 6.5y | 0  (HOMA-IR) | 0  (HOMA-IR) | No differences between groups (**adjusted for BMI**) |
| Uçar 2014^L^  (29) | B-6.5y  (WT gain) | 31 | 6.5y | ++  (OGTT) | ++  (OGTT) | SGA girls with premature adrenarche had higher IR than AGA (**adjusted for BMI**) |
| Evagelidou 2007^M^ (37) | B-7y  (WT gain) | 35 | 7y | 0  (HOMA-IR) |  | WT gain was not associated with IR |
| Ibañez 2009^M^  (39) | B-7y  (WT gain) | 56 | 7y | ++  (HOMA-IR) |  | WT gain was positively associated with IR |
| Bavdekar 1999^M^ (44) | B-8y  (WT gain) | 165 | 8y | ++  (HOMA-IR) | 0  (HOMA-IR) | WT gain was positively associated with IR |
| Torre 2008^M^  (31) | B-8y  (WT, HT, BMI gain) | 52 | 8y | 0  (HOMA-IR) | 0  (HOMA-IR) | Catch up growth was not associated with IR, if BMI was normal |
| Cianfarani 2003^M^ (17) | B-8.5y  (HT gain) | 82 | 8.5y | 0  (HOMA-IR) |  | No differences between groups (**adjusted for BMI**) |
| Veening 2003^M^  (16) | B-1y  B-2y  2-9y  (BMI gain) | 28 | 1y, 2y, 9y | 0 (0-2y, Clamp)  ++ (2-9y, Clamp) | ++ (Clamp) | BMI gain between 2-9y was positively associated with IR |
| Fabricius-Bjerre 2011^M^  (46) | B-3mo  B-1y  (WT gain) | 30 | 17.6y | ++  (HOMA-IR) | ++  (HOMA-IR) | WT gain was positively associated with IR (**adjusted for BMI**) |
| Leunissen 2008^H^ (41) | B-21.5y  (WT gain) | 71 | 21.5y | +  (IVGTT) | ++  (IVGTT) | Catch up growth was associated with higher IR (**adjusted for height and fat mass**) |

| **c) Blood pressure** | **Timing of exposure (nature)** | **N**  **SGA** | **Age at outcome** | **Summary result** | **SGA vs. AGA controls** | **Comment** |
| --- | --- | --- | --- | --- | --- | --- |
| **Randomised controlled trials** | | | | | | |
| Singhal 2007^H^ (21) | 4d-9mo  (Nutrient enriched formula) | 153 | 6-8y | ++  (BP) |  | Enriched formula increased the risk of high BP at 6-8 y WT gain B-9mo was positively associated with systolic BP |
| **Observational studies (4 studies)** | | | | | | |
| Hemachandra 2007^M^ (48) | B-4mo  4mo-1y  1-4y  4-7y  (WT gain) | 2802 | 7y | ++  (BP) | 0  (BP) | WT gain during any period was positively associated with high systolic BP |
| Bavdekar 1999^M^ (44) | B-8y  (WT gain) | 165 | 8y | ++  (BP) | +  (BP) | WT gain was associated with higher BP, TG and TChol |
| Horta 2003^M^  (49) | B-20mo,  B-42mo  (WT gain) | 38 | 15y | ++  (BP) | 0  (BP) | WT gain was associated with higher systolic BP |
| Leunissen 2012^M^ (42) | B-21y  (WT gain) | 106 | 21y | 0 (BP) | 0  (BP) | WT gain was not associated with BP |
| **d) Lipids**  **Observational studies – (7 studies)** | | | | | | |
| Soto 2003^M^  (15) | B-1y  (WT, HT gain) | 85 | 1y | 0  (TChol, TG) | +  (TG) | No association with blood lipids between SGA groups |
| Deng 2012^M^  (45) | B-6y  (WT gain) | 111 | 6y | 0  (TG) | 0  (TG) | No association with TG levels (adjusted for BMI) |
| Evagelidou 2007^M^ (37) | B-7y  (WT gain) | 35 | 7y | 0  (TChol, TG) | 0  (TCho, TG) | No association with blood lipids |
| Bavdekar 1999^M^ (44) | B-8y  (WT gain) | 165 | 8y | ++  (TChol, TG) | +  (TChol, LDL) | WT gain was associated with higher BP, TG and TChol |
| Torre 2008^M^  (31) | B-8y  (BMI, HT gain) | 52 | 8y | 0 (TChol, LDL & TG)  -- (HDL, HT gain) | 0  (LDL, HDL, TG) | HT gain was inversely associated with HDL cholesterol |
| Cianfarani 2003^M^ (17) | B-8.5y  (HT) | 82 | 8.5y | -- (TChol & LDL)  0 (TG & HDL) | --  (TChol, HDL) | HT gain was inversely associated with TChol & LDL |
| Tenhola 2000^M^ (50) | B-5y  (HT gain) | 55 | 12 y | --  (TChol) | ++  (TChol) | SGA with poor HT gain had higher TChol |

B, birth; BMI, body mass index; WT, weight; HT, height or length; IR, insulin resistance; SGA, small-for-gestational age; AGA, appropriate-for-gestational age; OGTT, oral glucose tolerance test; IVGTT, intravenous glucose tolerance test; BP; blood pressure; TG, triglycerides; TChol, total cholesterol; CIMT, carotid-intimal thickness; y, years; mo, months. (++) statistically significant positive association; (+) non-significant positive trend; (0) no association; (-) non-significant inverse trend; and (--) significant inverse association.
